# Estimating population infection rates from non-random testing data: Evidence from the COVID-19 pandemic

**DOI:** 10.1371/journal.pone.0311001

**Published:** 2024-09-26

**Authors:** David Benatia, Raphael Godefroy, Joshua Lewis

**Affiliations:** 1 Department of Applied Economics, HEC Montréal, Quebec, Canada; 2 Department of Economics, Université de Montréal, Quebec, Canada; International Institute of Information Technology, INDIA

## Abstract

To effectively respond to an emerging infectious disease outbreak, policymakers need timely and accurate measures of disease prevalence in the general population. This paper presents a new methodology to estimate real-time population infection rates from non-random testing data. The approach compares how the observed positivity rate varies with the size of the tested population and applies this gradient to infer total population infections. Applying this methodology to daily testing data across U.S. states during the first wave of the COVID-19 pandemic, we estimated widespread undiagnosed COVID-19 infections. Nationwide, we found that for every identified case, there were 12 population infections. Our prevalence estimates align with results from seroprevalence surveys, alternate approaches to measuring COVID-19 infections, and total excess mortality during the first wave of the pandemic.

## Introduction

Early and aggressive public health interventions can substantially reduce pandemic mortality. Evidence from both the Coronavirus 2019 (COVID-19) outbreak and the 1918–1919 Influenza Pandemic suggest that tens of thousands of lives were lost due to minor delays in the initial adoption of preventative public health measures [1–3]. To effectively respond in the early stages of an infectious disease outbreak, policymakers need timely and accurate information on local disease prevalence.

Our understanding of population infection rates may be limited by constraints on testing capabilities in the early stages of an outbreak. During the first wave of COVID-19, severe constraints on the supply of PCR tests in the U.S. meant that testing was limited to a small number of high-risk individuals, and many mild or asymptomatic cases went undiagnosed [4–6]. Moreover, the absence of randomized population-based testing makes it is impossible to infer population infection rate from the share of positive cases among the tested sample, since the selection of high-risk individuals into testing will lead the sample positivity rate to overstate disease prevalence in the overall population. Finally, wide differences in testing capabilities across both countries and subnational jurisdictions can hamper our understanding of geographic spread of the disease, since more cases will be identified in locations where testing is more widely available. Indeed, by early April 2000, South Korea had conducted three times more per capita COVID-19 tests than the U.S., while New York state had conducted nearly twice as many per capita tests as New Jersey [7, 8].

The main objective of this paper is to develop a methodology to estimate real-time population infection rates in the early stages of an infectious disease outbreak. The methodology corrects the observed positivity rates among tested individuals for non-random sampling to calculate overall population infection rates. The approach builds on insights from econometrics on the issue of sample selection bias [9–14], and can be used to estimate disease prevalence at various jurisdictional levels (national and subnational) based on widely available testing data. Further, the methodology does not require information on clinical or epidemiological characteristics of the disease, such as the case fatality rate, the asymptomatic proportion, or the reproductive number; factors over which there is often considerable uncertainty during the early phases of an outbreak [15–17].

The methodology is based on the insight that the relationship between the positivity rate and the size of the tested population can be used to assess the severity of selection bias. For example, a *negative* slope indicates *positive* selection bias, since individuals who are most frequently tested have the highest probability of infection. Once the functional form of this relationship is estimated, the population infection rate can be computed as a combination of the *observed* positivity rate and the *estimated* selection gradient, which corrects for non-random testing.

The second objective of this paper is to apply this methodology to daily data on COVID-19 testing rates and positivity rates across U.S. states from late March to early April 2020 to estimate population infection rates during the first wave of the pandemic. The key identification assumption for the analysis is that testing rates must be unrelated to underlying population disease prevalence. To ensure that this assumption is met, we focused on high frequency day-to-day variation in testing across states. Intuitively, because there is little scope for disease prevalence to evolve from one day to the next, daily changes in testing rates should be orthogonal to underlying changes in population infection rates. In addition, we estimated generalized versions of the model, that relax this identification assumption.

Finally, we assessed the validity of the methodology, by comparing the estimated population infection rates across states to alternate measures of pandemic severity during the first wave. These measure include 1) estimates of population prevalence for SARS-CoV-2 antibodies taken in a number of specific geographical sites in April and May 2020, 2) estimates of population COVID-19 infections by early April based on an alternative methodology that relies on retrospective COVID-19 deaths, and 3) total all-cause excess mortality during the first wave of the pandemic.

Our empirical framework complements existing methods used to estimate population infection rates in the United States and internationally [18–25]. One approach has been based on the Susceptible Infectious Recovered (SIR) epidemiological model, which calibrates parameters to the specific characteristics of the SARS-CoV-2 pandemic to estimate current and future infections. A challenge for this approach is the large uncertainty regarding the relevant parameter values for the virus, particularly in the early stages of an outbreak. Other research has relied on Bayesian modelling to infer past disease prevalence from observed COVID-19 deaths. These models require fewer assumptions regarding the underlying parameter values. However, given the extended delay between initial infection and death [26], estimates based on this approach will identify disease prevalence with a significant lag, so cannot be used to provide information of real-time infection rates.

Most closely related to our paper is [27], who use data on the total number of total tests and the positive test rate to estimate ranges for population COVID-19 infection rates for Illinois, New York, and Italy in early April. Their approach imposes only a weak monotonicity assumption for identification, but produces wide bounds on infection rates. Our approach produces much narrower intervals, but requires imposing some structure on the selection process. Policymakers should be aware that bounds that account for model uncertainty would be wider than ours, as is not uncommon in econometric analyses [28].

## Materials and methods

### Data

The main analysis was based on daily data on total test results (positive plus negative) and the positivity rate (positive tests divided by total tests) across U.S. states from March 31 to April 7. This period was selected to coincide with the sharp rise in reported cases in U.S. during the first wave, and for ease of comparison to a number of seroprevalence studies conducted around the same time period. We excluded earlier observation to limit errors associated with changes in state reporting practices throughout March, although research suggests that community transmission in many states was already widespread by mid-March [25].

The data were obtained from the COVID Tracking Project, a site launched by journalists from The Atlantic that publishes high-quality data on the outbreak across U.S. states [7]. The data were compiled primarily from state public health authorities, occasionally supplemented with information from news reporting, official press releases, or messages from officials released on Facebook or Twitter. We supplemented these data with information on total state population [29].

### Methods

#### Theory

A simple selection model for testing was developed to link the observed positivity rate among the sample of tested individuals to overall population infection rate.

We consider a stable population, normalized to size one, and denote *A* and *B* as the number of sick and healthy individuals, respectively. Let *p*_*n*_ denote the probability that a sick person is tested and *q*_*n*_ the probability that a healthy person is tested, given a total number of tests, *n*. Thus, we have:

$$n=p_{n}A+q_{n}B,$$

and assuming the test is accurate, the number of positive tests is:

$$s=p_{n}A.$$

This framework highlights how non-random testing will bias estimates of the population disease prevalence. Using Bayes’ rule, we can write the relative probability of testing as the following:

$$\frac{p_{n}}{q_{n}}=\frac{Pr(sick|tested,n)/Pr(healthy|tested,n)}{Pr(sick|n)/Pr(healthy|n)},$$

which is equal to one if tests are randomly allocated. When testing is targeted to individuals who are more likely to be sick, we have *Pr*(*sick*|*tested*, *n*) > *Pr*(*sick*|*n*) and *Pr*(*healthy*|*tested*, *n*) < *Pr*(*healthy*|*n*), so the ratio will be greater than one. In this scenario, the ratio of sick to healthy people in the sample, *p*_*n*_*A*/*q*_*n*_*B*, will exceed the ratio in the overall population, *A*/*B*.

We assume that the severity of selection bias can be expressed a function of the number of tests:

$$\begin{array}{c} \frac{p_{n}}{q_{n}}=f\left(n;\theta \right) \end{array}$$ (1)

where *n* is number of conducted tests and *θ* is a vector of parameters to be estimated.

According to this setup, the fraction of positive tests, *s*/*n*, can be written as follows:

$$\begin{array}{c} \frac{s}{n}=\frac{1}{1+\frac{q_{n}}{p_{n}}\frac{B}{A}} \end{array}$$ (2)

In practice, the denominator of Eq (2) is much larger than one—during the sample period, the median ratio of negative to positive tests, $\frac{q_{n}A}{p_{n}B}$, across U.S. states was 7.3. Thus, taking logs of Eq (2), we can make the following approximation:

$$\begin{array}{c} \text{log}\frac{s}{n}\approx \text{log}(\frac{p_{n}}{q_{n}}\frac{A}{B})=\text{log}(\frac{p_{n}}{q_{n}})+\text{log}(\frac{A}{B}) \end{array}$$ (3)

Eq (3) shows that the log share of positive tests in the sample can be approximated by the sum of the log ratio of the relative probability of testing, *p*_*n*_/*q*_*n*_, and the *unobserved* log ratio of sick to healthy people in the population, *A*/*B*.

#### From theory to estimation

To conduct the estimation, a first difference estimator was adopted, where the dependent variable is the difference $\text{log}\frac{s_{i,t}}{n_{i,t}}-\text{log}\frac{s_{i,t-1}}{n_{i,t-1}}$ on two consecutive days *t* − 1 and *t* in a given state *i*. Given the last equation, this first difference is equal to:

$$\begin{array}{c} \text{log}\frac{s_{i,t}}{n_{i,t}}-\text{log}\frac{s_{i,t-1}}{n_{i,t-1}}=\text{log}\;f\left(n_{i,t};\theta \right)-\text{log}\;f\left(n_{i,t-1};\theta \right)+u_{i,t} \end{array}$$ (4)

where $u_{i,t}=\text{log}(\frac{A_{i,t}}{B_{i,t}})-\text{log}(\frac{A_{i,t-1}}{B_{i,t-1}})+\epsilon_{i,t}$ is a mean zero error term that depends on the change in ratio of sick to healthy individuals in the population from *t* − 1 to *t* and an idiosyncratic component, *ϵ*_*i*,*t*_.

Eq (4) forms the basis of the empirical analysis. The identifying assumption is strict exogeneity in the error term: *E*(*u*_*i*,*t*_|*n*_*i*,*t*_, *n*_*i*,*t*−1_). This assumption ensures that the errors are uncorrelated with any function of changes in the number of tests, Δ*n*_*i*,*t*_, and will be violated if changes in the population infection rate are systematically related to testing capacity. This assumption is supported by the short time interval in the daily first difference specification, which limits the scope for disease evolution. In some specifications, we add controls for state fixed effects to allow for jurisdiction-specific exponential growth in underlying disease prevalence from one day to the next.

By focusing on a daily first difference estimator, we are able to partial out the unobserved log ratio of sick to healthy people in the population, *A*_*i*,*t*_/*B*_*i*,*t*_. As a result, changes in the positivity rate depend on the number of tests *only* through a selection channel.

Using day-to-day changes in the positivity rate and day-to-day changes in the number of tests, we can recover $\text{log}\;f(n;\hat{\theta })$ by estimating Eq (4). This term captures the predicted change in the positivity rate as a function of the number of tests, *n*. We can recover the estimated population infection rates, ${\hat{\frac{s_{pop}}{n_{pop}}}}_{i,t}$, as the estimated positivity rate if the entire population in state *i* were tested on date *t*, i.e. *n*_*i*,*t*_ = *pop*_*i*_.

The estimated population infection rate can be obtained by rewriting Eq (4) as:

$$\begin{array}{c} \text{log}{\hat{\frac{s_{pop}}{n_{pop}}}}_{i,t}=\text{log}\frac{s_{i,t}}{n_{i,t}}+\text{log}\;f\left(pop_{i};\hat{\theta }\right)-\text{log}\;f\left(n_{i,t};\hat{\theta }\right) \end{array}$$ (5)

Eq (5) shows that the estimated log population infection rate is equal to the *observed* log sample positivity rate, $\text{log}\frac{s_{i,t}}{n_{i,t}}$, plus an adjustment factor that corrects for non-random testing, $\text{log}\;f\left(pop_{i};\hat{\theta }\right)-\text{log}\;f(n_{i,t})$.

One could also view this exercise as a reduced form estimation of the relationship between the fraction of individuals who test positive and the size of the tested population, holding constant the population share of sick. Once this relationship has been consistently estimated, we can calculate the estimated share of positive tests for any value of *n*, including when *n* = *pop*_*i*_.

#### Empirical implementation

To implement the procedure, we specify the following functional form for the selection process into testing, *f*(*n*; *θ*):

$$\frac{p_{n}}{q_{n}}=f\left(n;\theta \right)=1+e^{\gamma +\beta n}.$$

The term *e*^*γ*+*βn*^ ≥ 0 reflects the fact that testing has been targeted towards higher risk populations, with the intercept, *γ*, capturing the severity of selection bias when testing is limited. Meanwhile, the coefficient *β* < 0 identifies how selection bias decreases with *n* as the ratio *p*_*n*_/*q*_*n*_ approaches one. Intuitively, as testing expands, the sample will become more representative of the overall population, and the selection bias will diminish.

Substituting this function into the first difference regression model and taking a third order power series approximation of the log function yields the following estimating equation:

$$\begin{array}{cc} \text{log}\frac{s_{i,t}}{n_{i,t}}-\text{log}\frac{s_{i,t-1}}{n_{i,t-1}} & =\alpha_{1}[e^{\beta \frac{n_{i,t}}{pop_{i}}}-e^{\beta \frac{n_{i,t-1}}{pop_{i}}}]+\alpha_{2}[e^{2\beta \frac{n_{i,t}}{pop_{i}}}-e^{2\beta \frac{n_{i,t-1}}{pop_{i}}}]\\ & +\alpha_{3}[e^{3\beta \frac{n_{i,t}}{pop_{i}}}-e^{3\beta \frac{n_{i,t-1}}{pop_{i}}}]+\nu_{i,t} \end{array}$$ (6)

where *ν*_*i*,*t*_ is the mean-zero Gaussian error term. Eq (6) forms the basis for the empirical analysis. The model was estimated by non-linear least squares, allowing for heteroskedastic errors.

Post-estimation, we derived predicted values for population infection rates based on Eq (5). The Delta method was used to approximate the standard errors of the estimated population infection rates. Specifically, the Delta method was used to calculate the standard errors of a first-order Taylor approximation of the function in Eq (5). The validity of this approach relies on the asymptotic normality and consistency of the parameter estimates, alongside the differentiability of the function of these parameters in Eq (5).

## Results

### Population COVID-19 infection rates by state

S1 Table reports the coefficients from Eq (6) estimated across states for the period March 31 to April 7. Model 1 reports the baseline estimates. Model 2 includes additional controls for state fixed effects. Model 3 excludes observations for which the state positivity rate was greater than 0.5. The coefficient estimates are broadly similar across the three specifications.

**Fig 1:**
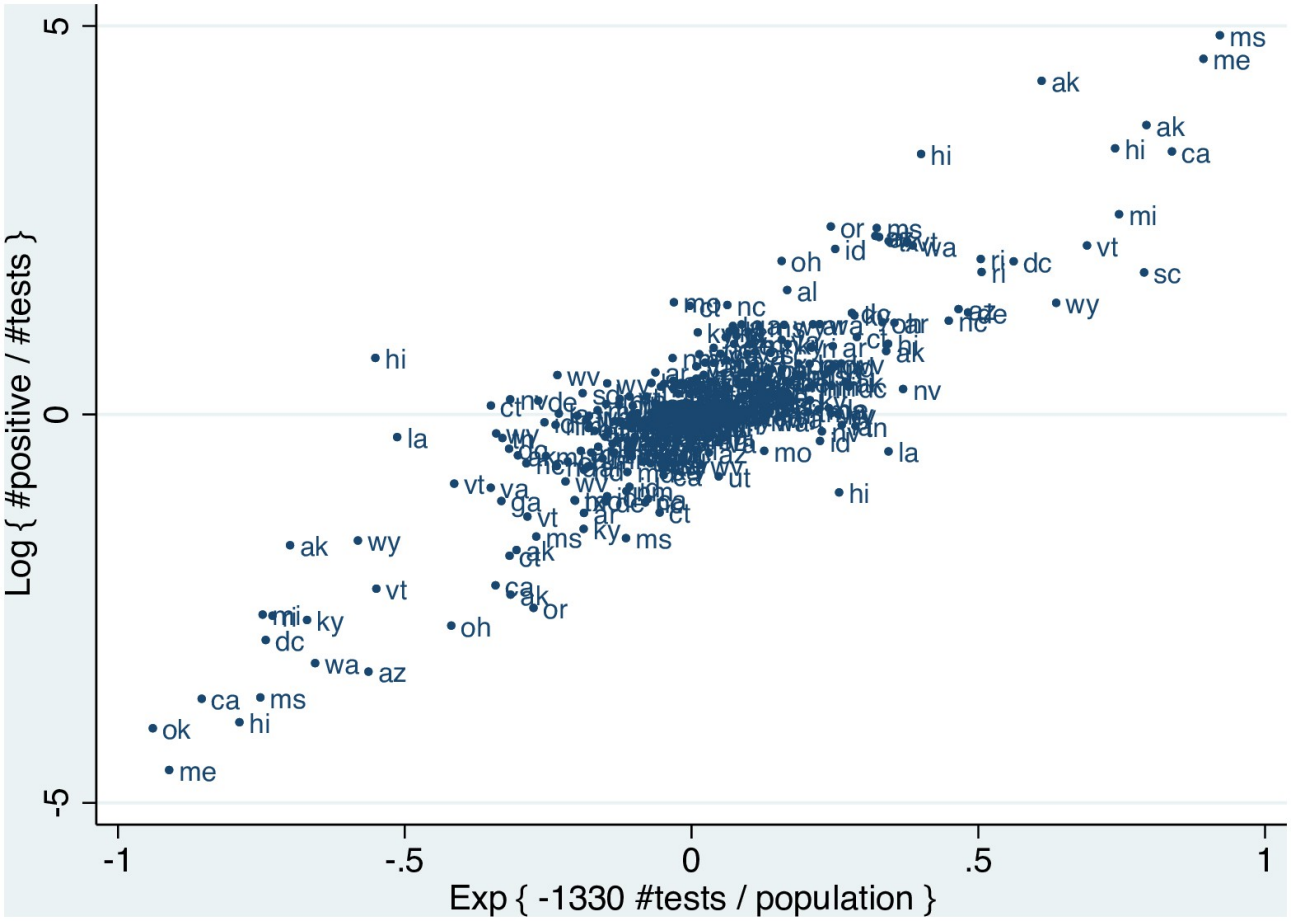
Daily changes in testing and the positivity rate, March 31—April 7, 2020. *Notes*: This figure reports the relationship between daily changes in the exponential of per capita testing and daily changes in the log positivity rate, using the coefficient of *β* derived from the main estimates of Eq (6).

Fig 1 depicts the relationship between daily changes in the positivity rate and per capita testing, based on the relationship implied by Eq (6). The linear empirical relationship indicates that the functional form of the model fits the data well. Because $\hat{\beta }$ is *negative*, the upward sloping pattern implies a negative relationship between daily changes in testing and the share of positive tests. A symptom of selection bias is that variables that have no structural relationship with the dependent variable may appear to be significant [10]. Thus, these patterns strongly suggest non-random testing, since daily changes in testing should be unrelated to population disease prevalence except through a selection channel.

Table 1 reports the results that adjust observed COVID-19 positivity rates for non-random testing based on the procedure described in the previous section. For reference, column (1) reports the observed positivity rate on April 7, 2020. Columns (2) and (3) report the adjusted rates for April 7 along with 95 percent confidence interval. Estimated population infection rates ranged from 0.3 percent in Wyoming to 7.6 percent in New Jersey. To put these estimates in perspective, in New York state, which had conducted the most extensive testing in the nation, 0.7 percent of the population had tested positive for COVID-19 by April 7. Our estimates imply that 34 states had population infection rates that were higher than the reported per capita cases in New York.

**Table 1:** Estimated population infection rates for COVID-19.

State	Positivity Rate on April 7 (%)	Estimated Population Infection Rate on April 7 (%)	95% Confidence Interval	Ave. Estimated Population Infection Rate, March 31—April 7 (%)
	(1)	(2)	(3)	(4)
AK	73.3	0.9	[0.5, 1.8]	0.4
AL*	10.2	1.0	[0.5, 2.1]	0.9
AR	9.0	0.7	[0.3, 1.5]	0.5
AZ	14.1	0.4	[0.2, 0.9]	0.6
CA	11.1	1.1	[0.5, 2.3]	0.9
CO	20.1	1.1	[0.5, 2.4]	1.8
CT	37.2	5.0	[2.4, 10.6]	4.2
DC	30.8	3.8	[1.8, 7.9]	3.0
DE**	15.2	1.9	[0.9, 3.9]	1.5
FL	9.6	1.3	[0.6, 2.8]	1.3
GA	61.7	4.2	[1.9, 9.1]	2.0
HI*	3.5	0.4	[0.2, 0.8]	0.4
IA	9.1	0.9	[0.4, 1.9]	0.7
ID	27.5	1.1	[0.5, 2.3]	1.5
IL	22.2	2.6	[1.2, 5.3]	2.3
IN	21.9	2.3	[1.1, 4.8]	1.9
KS	12.8	0.5	[0.2, 1.1]	0.7
KY	4.5	0.3	[0.2, 0.8]	0.4
LA	25.8	5.7	[2.5, 12.9]	6.7
MA	27.8	3.9	[1.8, 8.3]	3.4
MD	16.2	1.5	[0.7, 3.3]	1.7
ME***	1.0	0.5	[0.3, 0.9]	0.5
MI	56.8	5.1	[2.4, 10.8]	4.4
MN	7.3	0.4	[0.2, 0.9]	0.3
MO	14.8	1.4	[0.7, 3.1]	1.1
MS*	0.8	0.7	[0.7, 0.8]	1.1
MT	10.3	0.5	[0.2, 1.2]	0.5
NC	98.6	1.1	[0.6, 2.2]	0.6
ND	2.4	0.3	[0.2, 0.7]	0.5
NE	8.1	0.6	[0.3, 1.3]	0.6
NH	12.6	1.0	[0.5, 2.1]	1.2
NJ	56.0	7.6	[3.6, 16.1]	7.6
NM	2.3	0.6	[0.3, 1.3]	0.7
NV	13.3	1.2	[0.6, 2.6]	1.2
NY	42.5	7.5	[3.3, 17.1]	8.5
OH	13.5	0.8	[0.4, 1.8]	0.9
OK	1.4	1.0	[0.7, 1.4]	1.0
OR	5.4	0.4	[0.2, 1.0]	0.5
PA	21.3	2.7	[1.3, 5.7]	2.4
RI*	42.3	4.2	[2.0, 8.9]	2.4
SC**	19.9	0.7	[0.3, 1.5]	1.0
SD	12.9	1.1	[0.5, 2.3]	0.8
TN	6.1	0.9	[0.4, 2.0]	0.9
TX	30.0	0.9	[0.4, 2.0]	0.6
UT	5.0	0.5	[0.3, 1.1]	0.7
VA	11.0	1.3	[0.6, 2.7]	0.9
VT	6.5	1.0	[0.4, 2.1]	1.4
WA*	11.4	1.3	[0.6, 2.7]	1.4
WI	6.6	0.7	[0.3, 1.4]	0.9
WV	3.2	0.7	[0.3, 1.6]	0.4
WY	7.9	0.3	[0.1, 0.6]	0.7

*Notes*: Column (2) reports the estimates for the population infection rate for COVID-19 based on the BGL methodology. Column (3) reports 95% confidence intervals for the estimates based on heteroskedasticity robust standard errors. Column (4) reports the average estimates for population infection rates for COVID-19 from March 31 to April 7, 2020. In cases of incomplete testing data on April 7, state population prevalence is reported for the closest day:

* indicates prevalence on April 6,

** indicates prevalence on April 5, and

*** indicates prevalence on March 31.

Table 1, col. (4) reports the average estimated population prevalence for the period March 31 to April 7. These averages mitigate sampling error in the daily prevalence estimates, which depend on the observed share of positive tests on any particular day. The average estimates are similar to the April 7 estimates, albeit generally smaller in magnitude, suggesting continued spread of the disease in many states.

Table 2 reports the results from several robustness exercises. First, we estimated modified versions of Eq (6) that include state fixed effects according to the following specification:

$$\begin{array}{cc} \text{log}\frac{s_{i,t}}{n_{i,t}}-\text{log}\frac{s_{i,t-1}}{n_{i,t-1}} & =\alpha_{1}[e^{\beta \frac{n_{i,t}}{pop_{i}}}-e^{\beta \frac{n_{i,t-1}}{pop_{i}}}]+\alpha_{2}[e^{2\beta \frac{n_{i,t}}{pop_{i}}}-e^{2\beta \frac{n_{i,t-1}}{pop_{i}}}]\\ & +\alpha_{3}[e^{3\beta \frac{n_{i,t}}{pop_{i}}}-e^{3\beta \frac{n_{i,t-1}}{pop_{i}}}]+\lambda_{s}+\nu_{i,t} \end{array}$$ (7)

where the term λ_*s*_ denotes a vector of state fixed effects. These models allow for an exponential trend in infection rates, thereby addressing concerns that underlying disease prevalence may evolve from one day to the next. In these models, each state to have its own specific intercept to capture the fact that the trends may differ depending on the local conditions.

**Table 2:** Robustness exercises.

	Estimated COVID-19 Prevalence on April 7						Estimated COVID-19 Prevalence Average: March 31—April 7		
	Baseline estimates		Add state fixed effects		Restrict to days w. < 50% positive cases		Baseline estimates	Add state fixed effects	Restrict to days w. < 50% pos. cases
	Est. (1)	95% CI (2)	Est. (3)	95% CI (4)	Est. (5)	95% CI (6)	(7)	(8)	(9)
AK	0.9	[0.5, 1.8]	1.0	[0.6, 1.6]			0.4	0.5	0.3
AL*	1.0	[0.5, 2.1]	1.0	[0.6, 1.8]	0.8	[0.4, 1.9]	0.9	0.9	0.8
AR	0.7	[0.3, 1.5]	0.7	[0.4, 1.3]	0.6	[0.3, 1.3]	0.5	0.6	0.5
AZ	0.4	[0.2, 0.9]	0.5	[0.3, 0.8]	0.4	[0.2, 0.9]	0.6	0.6	0.5
CA	1.1	[0.5, 2.3]	1.1	[0.7, 2.0]	0.9	[0.4, 2.1]	0.9	0.9	0.8
CO	1.1	[0.5, 2.4]	1.2	[0.7, 2.1]	0.9	[0.4, 2.2]	1.8	1.9	1.5
CT	5.0	[2.4, 10.6]	5.2	[3.0, 9.1]	4.2	[1.8, 9.5]	4.2	4.3	3.1
DC	3.8	[1.8, 7.9]	3.9	[2.3, 6.7]	3.2	[1.4, 7.0]	3.0	3.1	2.4
DE**	1.9	[0.9, 3.9]	2.0	[1.1, 3.4]	1.6	[0.7, 3.5]	1.5	1.6	1.3
FL	1.3	[0.6, 2.8]	1.4	[0.8, 2.4]	1.1	[0.5, 2.5]	1.3	1.3	1.1
GA	4.2	[1.9, 9.1]	4.3	[2.5, 7.7]			2.0	2.1	1.4
HI*	0.4	[0.2, 0.8]	0.4	[0.2, 0.7]	0.3	[0.1, 0.7]	0.4	0.5	0.4
IA	0.9	[0.4, 1.9]	0.9	[0.5, 1.6]	0.8	[0.3, 1.7]	0.7	0.7	0.6
ID	1.1	[0.5, 2.3]	1.1	[0.6, 2.0]	0.9	[0.4, 2.1]	1.5	1.6	1.3
IL	2.6	[1.2, 5.3]	2.7	[1.6, 4.6]	2.1	[1.0, 4.8]	2.3	2.4	1.9
IN	2.3	[1.1, 4.8]	2.4	[1.4, 4.1]	1.9	[0.8, 4.3]	1.9	2.0	1.6
KS	0.5	[0.2, 1.1]	0.5	[0.3, 1.0]	0.4	[0.2, 1.0]	0.7	0.7	0.6
KY	0.3	[0.2, 0.8]	0.4	[0.2, 0.6]	0.3	[0.1, 0.7]	0.4	0.4	0.3
LA	5.7	[2.5, 12.9]	5.9	[3.2, 10.8]	4.6	[1.9, 11.3]	6.7	6.9	5.0
MA	3.9	[1.8, 8.3]	4.0	[2.3, 7.1]	3.2	[1.4, 7.3]	3.4	3.6	2.8
MD	1.5	[0.7, 3.3]	1.6	[0.9, 2.8]	1.3	[0.6, 2.9]	1.7	1.8	1.4
ME***	0.5	[0.3, 0.9]	0.5	[0.4, 0.8]	0.5	[0.2, 0.9]	0.5	0.5	0.5
MI	5.1	[2.4, 10.8]	5.3	[3.0, 9.2]			4.4	4.6	3.2
MN	0.4	[0.2, 0.9]	0.4	[0.3, 0.8]	0.4	[0.2, 0.8]	0.3	0.3	0.3
MO	1.4	[0.7, 3.1]	1.5	[0.9, 2.6]	1.2	[0.5, 2.7]	1.1	1.2	0.9
MS*	0.7	[0.7, 0.8]	0.7	[0.7, 0.8]	0.7	[0.7, 0.8]	1.1	1.1	0.9
MT	0.5	[0.2, 1.2]	0.6	[0.3, 1.0]	0.5	[0.2, 1.1]	0.5	0.5	0.4
NC	1.1	[0.6, 2.2]	1.2	0.7, 1.9			0.6	0.7	0.5
ND	0.3	[0.2, 0.7]	0.3	[0.2, 0.6]	0.3	[0.1, 0.6]	0.5	0.5	0.4
NE	0.6	[0.3, 1.3]	0.6	[0.3, 1.1]	0.5	[0.2, 1.1]	0.6	0.6	0.5
NH	1.0	[0.5, 2.1]	1.0	[0.6, 1.8]	0.8	[0.4, 1.9]	1.2	1.3	1.0
NJ	7.6	[3.6, 16.1]	7.9	[4.5, 13.8]			7.6	7.9	6.0
NM	0.6	[0.3, 1.3]	0.6	[0.3, 1.1]	0.5	[0.2, 1.1]	0.7	0.7	0.5
NV	1.2	[0.6, 2.6]	1.3	[0.7, 2.2]	1.0	[0.5, 2.4]	1.2	1.2	1.0
NY	7.5	[3.3, 17.1]	7.9	[4.3, 14.4]	6.2	[2.6, 14.9]	8.5	8.8	7.0
OH	0.8	[0.4, 1.8]	0.9	[0.5, 1.5]	0.7	[0.3, 1.6]	0.9	0.9	0.7
OK	1.0	[0.7, 1.4]	1.0	[0.8, 1.3]	0.9	[0.6, 1.4]	1.0	1.0	0.9
OR	0.4	[0.2, 1.0]	0.5	[0.3, 0.8]	0.4	[0.2, 0.9]	0.5	0.5	0.4
PA	2.7	[1.3, 5.7]	2.8	[1.6, 4.9]	2.3	[1.0, 5.1]	2.4	2.5	2.0
RI*	4.2	[2.0, 8.9]	4.4	[2.5, 7.6]	3.5	[1.6, 8.0]	2.4	2.5	2.0
SC**	0.7	[0.3, 1.5]	0.7	[0.4, 1.3]	0.6	[0.3, 1.4]	1.0	1.0	0.9
SD	1.1	[0.5, 2.3]	1.1	[0.6, 1.9]	0.9	[0.4, 2.0]	0.8	0.8	0.7
TN	0.9	[0.4, 2.0]	1.0	[0.5, 1.7]	0.8	[0.3, 1.8]	0.9	1.0	0.8
TX	0.9	[0.4, 2.0]	1.0	[0.5, 1.7]	0.8	[0.3, 1.8]	0.6	0.7	0.6
UT	0.5	[0.3, 1.1]	0.6	[0.3, 1.0]	0.4	[0.2, 1.0]	0.7	0.7	0.6
VA	1.3	[0.6, 2.7]	1.4	[0.8, 2.3]	1.1	[0.5, 2.4]	0.9	1.0	0.8
VT	1.0	[0.4, 2.1]	1.0	[0.6, 1.8]	0.8	[0.3, 1.8]	1.4	1.5	1.2
WA*	1.3	[0.6, 2.7]	1.4	[0.8, 2.3]	1.1	[0.5, 2.4]	1.4	1.4	1.1
WI	0.7	[0.3, 1.4]	0.7	[0.4, 1.2]	0.6	[0.2, 1.3]	0.9	0.9	0.7
WV	0.7	[0.3, 1.6]	0.7	[0.4, 1.3]	0.6	[0.2, 1.4]	0.4	0.4	0.4
WY	0.3	[0.1, 0.6]	0.3	[0.2, 0.5]	0.2	[0.1, 0.6]	0.7	0.7	0.6

*Notes*: Columns (1) to (6) report the estimates and heteroskedasticity robust 95% confidence intervals for population prevalence of COVID-19 on April 7 based on the BGL methodology. Columns (7) to (9) report the the average estimates for population prevalence of COVID-19 from March 31 to April 7. Columns (3), (4) and (8) report results based on models that include state fixed effects. Columns (5), (6), and (9) report results based on models that restrict the sample to observations for which the share of positive cases was less than 0.5. In cases of incomplete testing data on April 7, population prevalence is reported for the closest day:

* indicates prevalence on April 6,

** indicates prevalence on April 5, and

*** indicates prevalence on March 31.

The results from these models (reported in cols. 2 and 7) are virtually identical to the baseline estimates. Moreover, the augmented model tends to produce more precise confidence intervals.

We also explored the sensitivity of the results to excluding observations with particularly high positivity rates. This specification addresses concerns regarding the functional form approximation made Eq (3) may not hold when $\frac{q_{n}A}{p_{n}B}$ is small. We restricted the sample to observations with a positivity rate below 0.5 and re-estimated Eq (6). Table 2, cols. 5,6,9 report the results. Although the sample size is reduced, the predicted infection rates are similar in magnitude to the baseline estimates and have similar confidence intervals.

### Comparison of BGL estimated population infection rates to alternative measures COVID-19 prevalence

To assess the validity of the methodology, we compared our state-level estimates of population infection rates (BGL methodology) to alternative measures of COVID-19 severity during the first wave of the pandemic. These alternative measures include 1) SARS-CoV-2 antibody prevalence from around the same time period; 2) estimated state-level COVID-19 infections based on an alternative methodology; and 3) excess mortality rates across states during the first wave.

The first set of comparisons are based on SARS-CoV-2 prevalence from population-based serological testing was conducted in a number of jurisdictions through the middle and end of spring wave (see S2 Table). Given the rapid upsurge in COVID-19 cases in late March, our estimates of current population prevalence in early April should be comparable to seroprevalence rates later in the month. These seroprevalence estimates thus provide a way to externally validate our estimated population infection rates. To expand the set of comparison localities, we also report our estimates of population infection rates for Ontario and Quebec based on testing data from [30], and compare these estimates to province-wide seroprevalence rates based on the same methodology applied to Canadian data [31].

**Fig 2:**
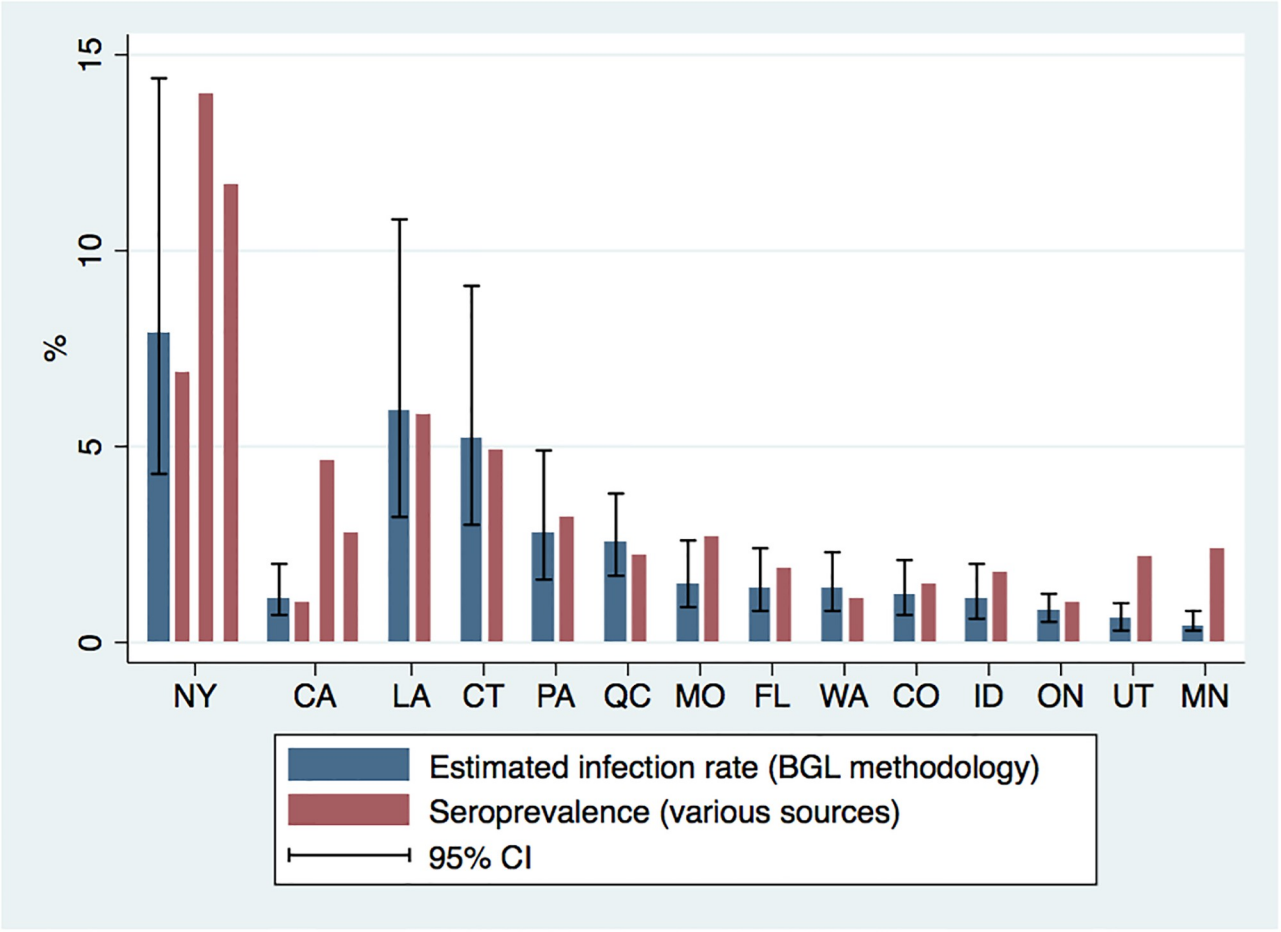
Estimates of population COVID-19 infection rates on April 7 and percent reactive to SARS-CoV-2 antibodies across 14 jurisdictions. *Notes*: This figure reports the estimates and 95% confidence intervals for population infection rates across states on April 7 based on BGL methodology, and estimates for SARS-CoV-2 seroprevalence from various sources (see S2 Table for details).

Fig 2 reports the estimates of population infection rates based on our methodology along with various estimates of the prevalence of SARS-CoV-2 antibodies across a number of geographical sites. There is broad similarity between the two prevalence estimates, and both approaches show evidence of widespread undetected infection during the first wave. The median difference is estimated prevalence is 23 percent, and the correlation between the sets of estimates is 0.88. The largest discrepancy between the two measures is in Minnesota, which experienced a sharp increase in COVID-19 cases between the time of our sample period (April 7) and the dates of specimen collection (April 30—May 12).

Next, we compared our results to estimated COVID-19 infections across U.S. states based on an alternative methodology: the Retrospective Methodology to Estimate Daily Infections from Deaths (REMEDID) [25]. The REMEDID approach reconstructs the time series of COVID-19 infections across U.S. states by combining information on the timing of state COVID-19 deaths with seroprevalence estimates taken later in the summer of 2020. Notably, this approach is based on an entirely different methodology and data sources than those from the BGL methodology. We compared the estimated population infection rates from the BGL methodology to the total number of COVID-19 infections across states through April 7 based on the REMEDID approach.

**Fig 3:**
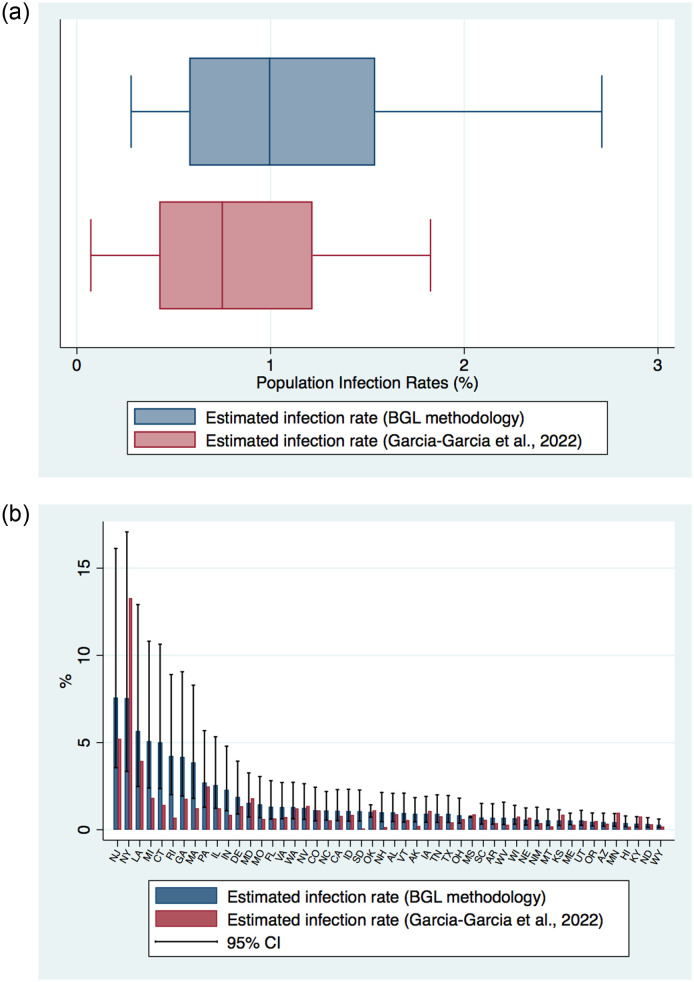
Estimated population COVID-19 infection rates across states based on the BGL and REMEDID methodologies. *Notes*: (a) This figure presents a box-plot of the distribution of estimated population COVID-19 infections across U.S. states based on the BGL and REMEDID methodologies [25]. Estimates for the REMEDID methodology are based on total estimated COVID-19 through to April 7, 2020. (b) This figure reports the estimated population COVID-19 infections based on the BGL and REMEDID methodologies across states, along with the 95% confidence interval.

Fig 3(a) reports box plots of the distribution of estimated population COVID-19 infection rates across states based on the two different methodologies. Both approaches yield similar estimates of total population infections, albeit slightly higher based on the BGL methodology. Median infection rates are 1.0 based on the BGL methodology and 0.75 based on the REMEDID approach. The distribution of COVID-19 infections are also similar, with a 25 to 75 percentile range of 0.6–1.5 and 0.4–1.2 for each methodology, respectively.

Fig 3(b) reports the estimated infection rates across states based on the BGL and REMEDID approaches. There is a close link between the two approaches. Indeed, the cross-state correlation in infection rates between the two different estimate approaches is 0.75.

**Fig 4:**
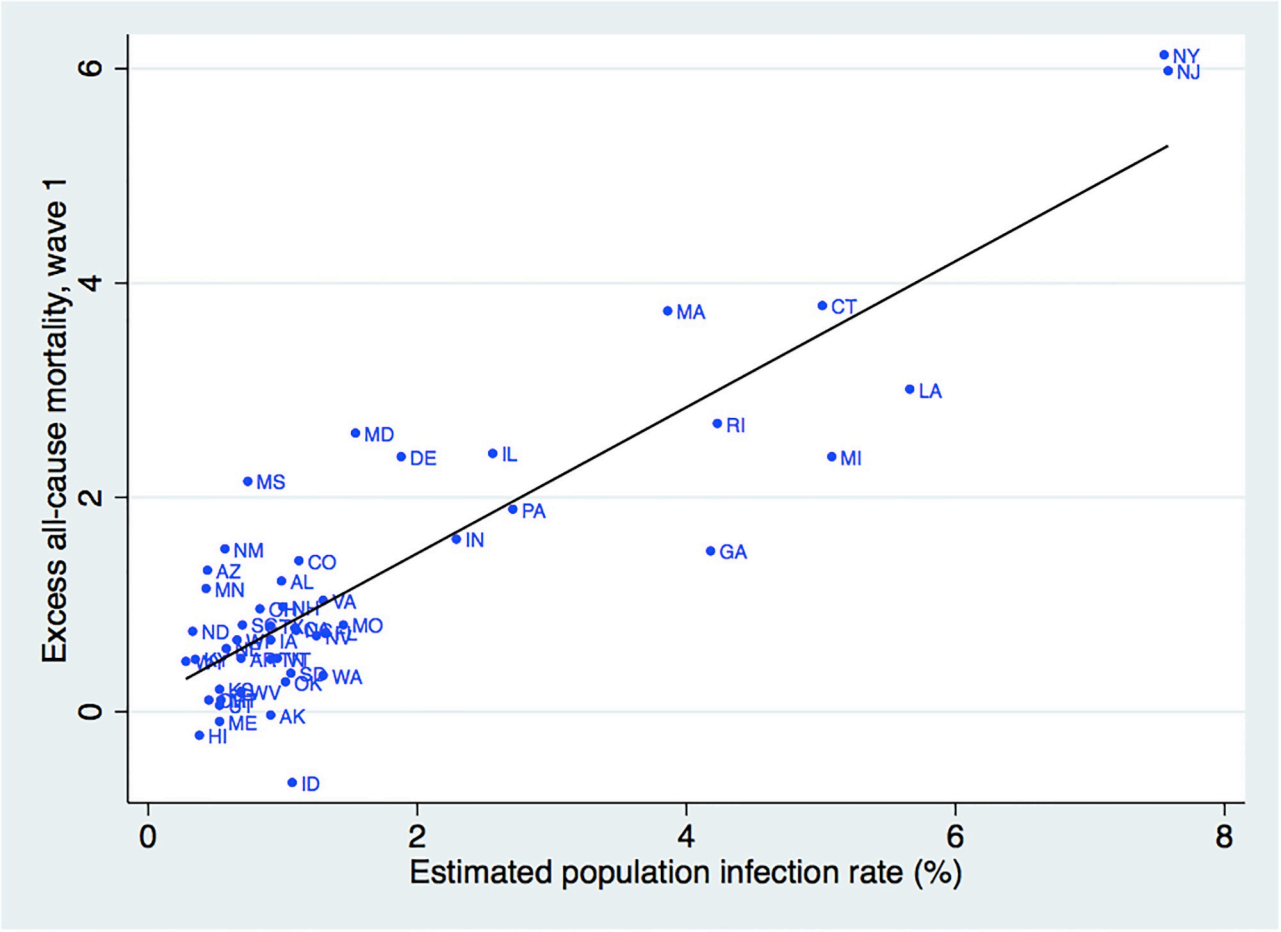
Estimated population COVID-19 infection rates and excess all-cause mortality (April 1 to June 30, 2020). *Notes*: This figure reports the relationship between estimated population COVID-19 infection rates on April 7, 2020 (based on the BGL methodology) and excess all-cause mortality from April 1 to June 30, 2020 [32]. The figure reports the bivariate scatterplot along with the best fit line.

Finally, we compared the BGL estimated infection rates to estimates of excess all-cause mortality across states during the first wave (April 1 through June 30, 2020) [32]. Fig 4 reports the scatterplot of these two measures, along with the best-fit line. There is a strong positive relationship between our estimates of COVID-19 and excess mortality. The correlation between the two measures is 0.88. Notably, every state that experienced excess mortality above 1.5 percent fell within the top quartile of estimated infection rates based on the BGL methodology. In contrast, all but two state with excess mortality below 1.5 fell in the bottom three quartiles of BGL estimated infections.

Together, these findings demonstrate a broad alignment between estimated population infection rates based on the BGL methodology and measures of overall COVID-19 prevalence during the first wave across based on alternative approaches.

### Population COVID-19 infection rates and state testing

Table 3 reports the relationship between the number of diagnosed cases and total population COVID-19 infections implied by our estimation procedure. We compared the average population infection rates from March 31 to April 7 to the total number of diagnosed cases by April 12. Because many individuals may not seek testing until the onset of symptoms, the latter date was chosen to correspond with the virus’s median incubation period [33, 34], although the delay between infection and symptom onset varies across individuals according to a lognormal distribution [35]. Column (1) reports the total diagnosed cases by April 12; column (2) reports the total number of COVID-19 cases implied by the estimates reported in Table 1 (col. 4); and column (3) presents the ratio of total cases to diagnosed cases.

**Table 3:** Diagnosed cases and estimated total cases of COVID-19.

State	Positive COVID-19 Tests, by April 12	Estimated Total COVID-19 Cases	Ratio of Total Cases to Positive Tests (2)/(1)	COVID-19 Tests per 1,000 Population
	(1)	(2)	(3)	(4)
AK	272	3,177	11.7	11.0
AL	3,525	44,155	12.5	4.4
AR	1,280	16,460	12.9	6.5
AZ	3,539	45,434	12.8	5.8
CA	21,794	353,000	16.2	4.8
CO	6,893	106,505	15.5	6.1
CT	12,035	148,252	12.3	11.6
DC	1,875	20,843	11.1	15.1
DE	1,479	14,779	10.0	11.4
FL	19,355	274,117	14.2	8.5
GA	12,452	215,306	17.3	5.1
HI	486	6,179	12.7	12.7
IA	1,587	22,678	14.3	5.6
ID	1,407	27,105	19.3	8.0
IL	20,852	287,087	13.8	7.9
IN	7,928	128,568	16.2	6.3
KS	1,337	19,110	14.3	4.5
KY	1,840	18,328	10.0	5.5
LA	20,595	310,465	15.1	22.4
MA	25,475	236,752	9.3	16.8
MD	8,225	102,114	12.4	8.2
ME	633	7,165	11.3	5.0
MI	24,638	441,486	17.9	8.0
MN	1,621	18,007	11.1	6.6
MO	4,160	69,549	16.7	7.4
MS	2,781	32,330	11.6	7.2
MT	387	5,138	13.3	8.3
NC	4,520	66,830	14.8	5.9
ND	308	3,507	11.4	13.6
NE	791	10,821	13.7	5.5
NH	929	16,792	18.1	8.0
NJ	61,850	672,314	10.9	14.3
NM	1,174	13,696	11.7	13.7
NV	2,836	36,864	13.0	8.0
NY	188,694	1,644,119	8.7	23.7
OH	6,604	100,221	15.2	5.4
OK	1,970	38,186	19.4	5.8
OR	1,527	21,994	14.4	7.1
PA	22,833	302,535	13.2	9.8
RI	2,665	25,081	9.4	19.2
SC	3,319	51,737	15.6	6.1
SD	730	7,205	9.9	9.7
TN	5,308	64,366	12.1	10.3
TX	13,484	187,963	13.9	4.3
UT	2,303	22,403	9.7	13.8
VA	5,274	80,574	15.3	4.7
VT	727	8,867	12.2	15.8
WA	10,224	103,188	10.1	12.3
WI	3,341	50,662	15.2	6.7
WV	611	7,724	12.6	9.1
WY	261	3,921	15.0	9.4

*Notes*: Columns (1) reports the cumulative number of positive COVID-19 tests by April 12. Column (2) reports the total number of COVID-19 cases implied by the average estimated population prevalence from March 31 to April 7 (Table 2, col. 4). Column (4) reports the cumulative number of COVID-19 tests by April 12 per 1,000 population.

The results reveal widespread undetected population infection. For every identified case nationwide, there were an estimated 12 total infections in the population. There were significant cross-state differences in these ratios. In New York, where more than two percent of the population had been tested, the ratio of total cases to positive diagnoses was 8.7, the lowest in the nation. Meanwhile, Oklahoma had the highest ratio in the country (19.4), and tested less than 0.6 percent of its population.

**Fig 5:**
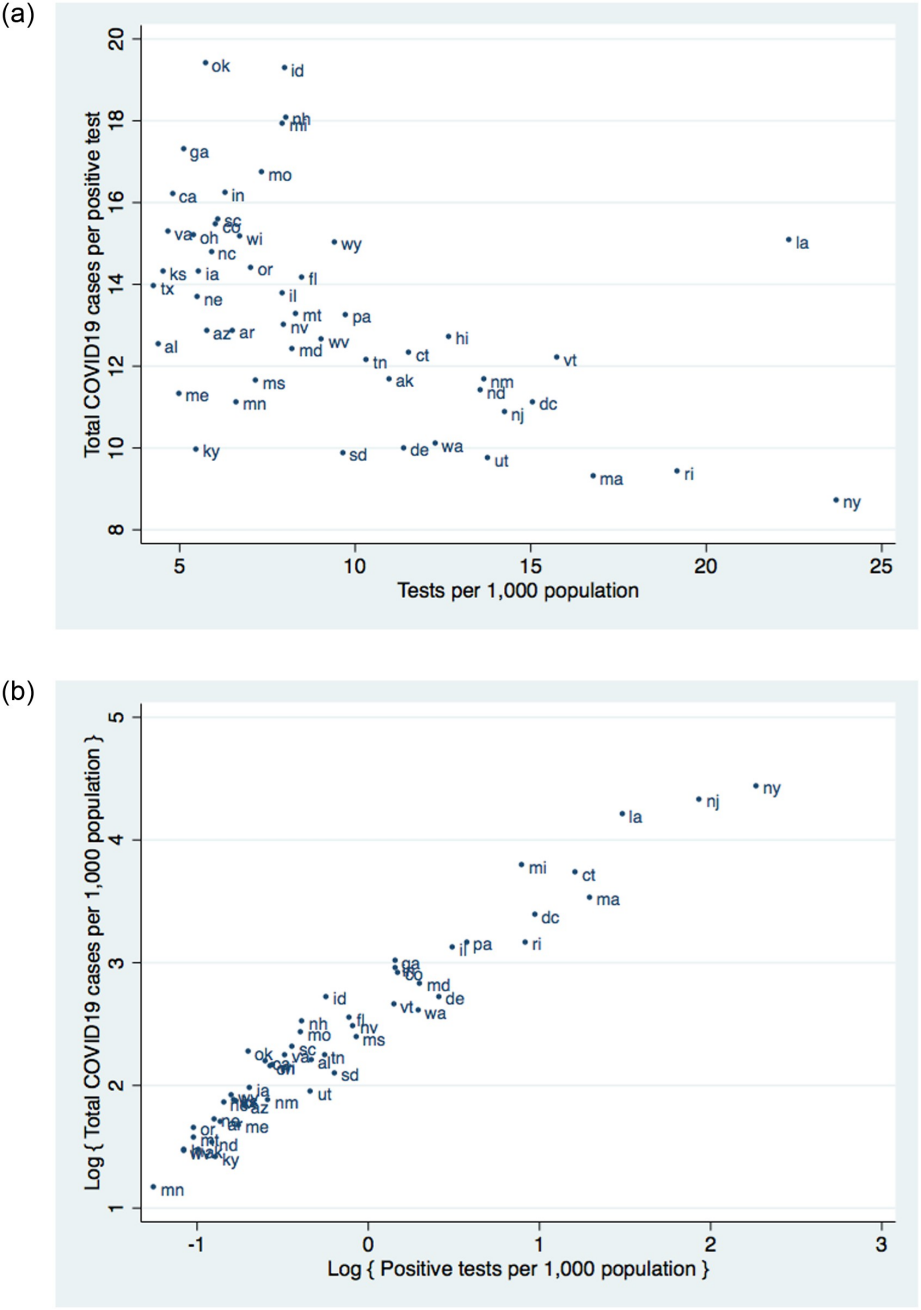
Testing and estimated population COVID-19 infection rates across states. *Notes*: (a) This figure presents the bivariate relationship between per capita testing and the ratio of total COVID-19 cases per diagnosis. Tests per 1,000 population are based on the cumulative number of tests by April 12. The ratio is the total number of COVID-19 cases, derived from the average estimated population prevalence from March 31 to April 7, divided by the cumulative number of positive tests by April 12. (b) This figure presents the bivariate relationship between log positive tests per capita and log total COVID-19 cases per capita. Positive tests per 1,000 population are based on the cumulative number of positive tests by April 12. The total number of COVID-19 cases is derived from the average estimated population prevalence from March 31 to April 7.

Fig 5(a) presents a bivariate scatter plot between the ratio of total COVID-19 cases per diagnosis and cumulative per capita testing by April 12. The negative relationship (corr = -0.51) indicates that relative differences in state testing do not simply reflect a response to geographic differences in pandemic severity. Instead, the patterns suggest that states that expanded testing capacity more broadly were better able to track population infections.

Fig 5(b) documents a positive relationship between per capita COVID-19 diagnoses and the estimated population infection rate. There is a strong positive relationship between the two series. Nevertheless, observed case counts do not perfectly predict overall population infections. For example, despite similar rates of reported COVID-19 cases, we find that Michigan had roughly twice as many per capita infections as Rhode Island. These differences can partly be explained by the fact that nearly two percent of the population in Rhode Island had been tested by April 12, whereas fewer than one percent had been tested in Michigan. Together, these findings suggest that differences in state-level policies towards COVID-19 testing may mask important differences in underlying disease prevalence.

## Conclusion

### Discussion and implications

This paper presents a new methodology to estimate population infection rates from non-random testing data. We applied this methodology to daily testing data for COVID-19 to estimate population infection rates across U.S. states during the first wave of the pandemic. We found widespread undocumented population infection. Our estimated infection rates are similar to findings from seroprevalence studies, retrospective estimates of total cases based on COVID-19 deaths, and all-cause excess mortality during the first wave. We also found that undetected infections were particularly high in states with lower testing capacity.

Pandemics pose an ongoing threat to public health. To effectively respond to these crises, policymakers need to have access to timely and accurate information on total population infections. Nevertheless, in the early phases of an infectious disease outbreak there is often considerable uncertainty regarding the extent of community transmission, as well as geographic spread of the disease.

In this paper, we have developed an approach that can be used to estimate real-time population infection rates from non-random test data. The estimation procedure is straightforward, has few data requirements, and can be used to estimate disease prevalence at various jurisdictional levels.

This methodology provides a useful tool for policymakers to track the scope of population infections during an emerging outbreak. Had the approach been applied to earlier testing data in March 2020, it would have revealed widespread undocumented community transmission that were only later confirmed by analyses of COVID-19 mortality and seroprevalence surveys. This information may have led policymakers to enact earlier and more aggressive public health interventions. Indeed, in an address to the House of Commons on June 10, 2020, Imperial College London epidemiologist Neil Ferguson stated that in March experts had “underestimated how far this country was into the epidemic,” and that “had we introduced lockdowns a week earlier we’d have reduced the final death toll by at least half. The measures, given what we knew about the virus then, were warranted. Certainly had we introduced them earlier we’d have seen many few deaths” [36].

The methodology could also be useful in guiding policymakers in how to allocate scarce health resources across jurisdictions. During the first wave of COVID-19, governments faced challenges in addressing shortages of health workers, personal protective equipment, and other health infrastructure [37]. At the same time, there was considerable uncertainty about the needs for these resources across localities. For example, by late February, community transmission was documented in Washington state, New York city, and Santa Clara county in California [38–40]. Nevertheless, of these three states in which COVID-19 was detected early, only New York experienced the dramatic surge in excess mortality during the first wave (Fig 4). Even within regions, idiosyncratic factors led to widespread variability in the severity of the first wave across jurisdictions. For example, the timing of Mardi Gras festival in late February led to a surge of COVID-19 cases in Louisiana that was far higher than those in other southern states [41]. By providing timely cross-jurisdiction information on population infection rates, our methodology could have enabled federal policymakers to better allocate scarce medical resources during the early onset of the pandemic.

### Policy implementation

To apply the BGL methodology, policymakers should adopt the following procedure:

1. Assemble high-frequency data on the total number of tests and the positivity rate across various geographic units.
2. Use these data to estimate Eq (7) by non-linear least squares.
3. Assess the functional form of the selection process by plotting the changes in the daily positivity rate against the daily change in exponential in per capita testing (as in Fig 1). If the specified function does not fit the data, consider alternative functional forms (linear, log linear) for the selection process, *f*(*n*, *θ*), and repeat step 2.
4. Combine the estimates from step 2 in Eq (5) to construct predicted values for population infection rates across each geographic unit and date.
5. Apply the Delta Method to Eq (5) to derive standard errors for the predicted population infection rates.

The results from this procedure can provide policymakers with estimates of disease prevalence across different geographic units during an emerging outbreak. For established infectious diseases, this approach can also be used to track the evolution of population disease prevalence over an extended time horizon.

### Limitations and future directions

There are four main limitations to our study which should be taken into account when interpreting the findings or applying the BGL methodology to track disease prevalence.

1. Errors in diagnostic testing results will not affect the accuracy of the predicted population infection rates but may reduce their precision.
2. Differing testing protocols across jurisdictions may reduce the accuracy of the predicted population infection rates.
3. Extended periods of latency between initial infection and symptom onset limit the use of the methodology to track real-time infection.
4. The accuracy of the predicted population infection rates depends on correctly specifying the functional relationship between the positive test rate and the size of the tested sample, over which there may be considerable uncertainty.

In what follows, we discuss each of these limitations in greater detail.

First, our analysis depends on the quality of diagnostic testing [42–44]. Because our analysis was focused on day-to-day variation, however, systematic false negative or false positive testing *will not* affect the estimates of population disease prevalence. This is because these errors are eliminated in the first difference Eq (6), provided that the rates of systematic testing errors are similar from one day to the next. Instead, systematic testing errors may reduce precision through classical measurement error [45], increasing the confidence intervals and leading to greater uncertainty in the true population infection rate.

Second, our approach requires an assumption that the underlying sample selection process was similar across observations. In practice, this assumption requires that decisions regarding how to prioritize tests were similar across jurisdictions. In our context this assumption is reasonable, given the short time span of the analysis and the fact that U.S. states faced a common set of guidelines for testing prioritization laid out by the CDC [46]. However, some caution should be taken when applying this approach to other contexts, such as analyses across countries with widely differing testing policies or extended time-series studies across multiple testing regimes, where observational units are highly dissimilar in their test allocation decisions.

Third, the ability of the methodology to track real-time infections is limited by the delay between infection and diagnosis, given that pre-symptomatic individuals are unlikely to seek testing. In the context of COVID-19, the median delay between initial infection and symptom onset has been estimated to be roughly 5 days [25, 47]. Despite this lag, the BGL methodology still provides timelier information on population infections than alternative methodologies based on COVID-19 deaths, which can only provide information on infections with an additional 2 to 8 week lag, given the extended delay between reported COVID-19 cases and mortality [26, 47, 48].

Finally, the estimates of population infection rates depend on a correctly specified functional relationship between the positive test rate and the size of the tested sample. In our empirical implementation, we specify a flexible functional relationship that fits the data well. Nevertheless, an important assumption underlying our analysis is that this observed relationship in the tested sample would continue to hold if testing were expanded out to the broader population. Future research might explore how to relax these functional form assumptions through either semi- or non-parametric approaches.

## Supporting information

S1 Table Coefficient estimates from Eq (6). (PDF)

S2 Table Date, location, and source for seroprevalence estimates. (PDF)

S1 Data Supplementary data. This zip file contains the underlying and analyzed datasets used to construct the tables and figures in the manuscript.
(ZIP)
