# Supplementary material for: Estimating population infection rates from non-random testing data: Evidence from the COVID-19 pandemic
Source: PLoS One. 2024 Sep 26;19(9):e0311001. doi: 10.1371/journal.pone.0311001 (PMC11426536; doi:10.1371/journal.pone.0311001)
Supplement: S1 Table — (PDF) [file pone.0311001.s001.pdf]

**Table S1.** Coefficient Estimates from Equation (6)

| | Model 1 | Model 2 | Model 3 |
|---|---|---|---|
| $\alpha_1$ | 11.1222<br>(1.9803) | 10.8495<br>(1.448) | 11.8478<br>(2.1104) |
| $\alpha_2$ | -21.6322<br>(3.765) | -21.0819<br>(2.754) | -22.6333<br>(3.8998) |
| $\alpha_3$ | 15.6053<br>(2.1573) | 15.2766<br>(1.5794) | 15.8989<br>(2.1975) |
| $\beta$ | -1330.7719<br>(167.8049) | -1336.3753<br>(126.3258) | -1242.6423<br>(157.7954) |
| $\sigma_u$ | 0.48136<br>(0.017984) | 0.4773<br>(0.01261) | 0.47424<br>(0.01837) |
| State fixed effects | | Yes | |
| Restrict to days with $< 50\%$ positive cases | | | Yes |
| Observations | 360 | 360 | 335 |

*Notes:* This table reports the estimation of the coefficients from equation (6). Model 1 presents the baseline results for the full sample. Model 2 reports the results with additional state fixed effects controls. Model 3 restricts the sample to observations for which the positivity rate was less than 0.5. Heteroskedasticity robust standard errors are reported in parentheses.
