# Supplementary material for: Estimating population infection rates from non-random testing data: Evidence from the COVID-19 pandemic
Source: PLoS One. 2024 Sep 26;19(9):e0311001. doi: 10.1371/journal.pone.0311001 (PMC11426536; doi:10.1371/journal.pone.0311001)
Supplement: S2 Table — (PDF) [file pone.0311001.s002.pdf]

Table S2. Date, Location, and Source for Seroprevalence Estimates

State	Estimate (95% CI)	Date of Specimen Collection	Location	Source
NY	6.9 (5.0 – 8.9)	Mar 23 – Apr 1	NYC metro area	[1]
NY	14.0 (13.3 – 14.7)	Apr 19 – 28	Statewide	[2]
NY	11.7	Apr 5 – 12	NYC (Mount Sinai hospital)	[3]
CA	1.0 (0.3 – 2.4)	April 23 – 27	San Francisco Bay area	[1]
CA	4.65 (2.52 – 7.07)	April 10 – 11	Los Angeles County	[4]
CA	2.8 (1.3 – 4.7)	April 3 – 4	Santa Clara County	[5]
LA	5.8 (3.9 – 8.2)	Apr 1 – 8	Statewide	[1]
CT	4.9 (3.6 – 6.5)	April 26 – May 3	Statewide	[1]
PA	3.2 (1.7 – 5.2)	Apr 13 – 25	Philadelphia metro area	[1]
QC	2.23	May 25 – July 9	Province wide (age 18 – 69)	[6]
MO	2.7 (1.7 – 3.9)	April 20 – 26	Statewide	[1]
FL	1.9 (1.0 – 3.2)	Apr 5 – 10	South Florida	[1]
WA	1.1 (0.7 – 1.9)	Mar 23 – Apr 1	Western Washington State	[1]
CO	1.5 (0.6 – 2.4)*	Mar 26 – Apr 7	San Miguel County	[7]
ID	1.79	Late April	Boise metro area	[8]
ON	1.1 (0.8 – 1.3)	June 5 – 30	Province wide	[9]
UT	2.2 (1.2 – 3.4)	April 20 – May 3	Statewide (age >18)	[1]
MN	2.4 (1.0 – 4.5)	April 30 – May 12	Minneapolis - St Paul - St Cloud metro area	[1]

Notes: This table describes the date, location and sources for the seroprevalence estimates, along with the 95% confidence interval (if reported). For San Miguel County, the reported range is based on two different estimates depending on whether "borderline" cases are included among positive tests.

Sources:

[1] Havers, F. P., C. Reed, T. Lim, J. M. Montgomery, J. D. Klena, et al. 2020. "Seroprevalence of Antibodies to SARS-CoV-2 in 10 Sites in the United States, March 23-May 12, 2020." *JAMA Internal Medicine*. DOI: 10.1001/jamainternmed.2020.4130.

[2] Rosenberg, E. S., J. M. Tesoriero, E. M. Rosenthal, R. Chung, M. A. Barranco, et al. 2020. "Cumulative Incidence and Diagnosis of SARS-CoV-2 Infection in New York." *Annals of Epidemiology*. 48: 23-29.

[3] Stadlbauer, D., J. Tan, K. Jiang, M. M. Hernandez, S. Fabre, et al. 2020. "Seroconversion of a City: Longitudinal Monitoring of SARS-CoV-2 Seroprevalence in New York City." medRxiv Working Paper. DOI:10.1101/2020.06.28.20142190.

[4] Sood, N., P. Simon, P. Ebner, et al. 2020. "Seroprevalence of SARS-CoV-2-Specific Antibodies Among Adults in Los Angeles County, California, on April 10-11, 2020." *JAMA*. 323(23): 2425-2427.

[5] Bendavid, E., B. Mulaney, N. Sood, et al. 2020. "COVID-19 Antibody Seroprevalence in Santa Clara County, California." medRxiv Working Paper. DOI: 10.1101/2020.04.14.20062463.

[6] Hema Quebec. Blood Donor Seroprevalence Study: 2.23% of Quebec Adults Contracted COVID-19. <https://www.hema-quebec.qc.ca/publications/communiques/archives/2020/communiques-2020/etude-seroprevalence-resultats.en.html> Accessed 2020-08-08.

[7] Klemko, R. Telluride Tested Nearly Everyone for Coronavirus. But the Crisis in New York Delayed the Results, and Their Usefulness. Washington Post. <https://www.washingtonpost.com/national/coronavirus-testing-telluride-colorado/2020/04/27/87e2f932-8624-11ea-878a-86477a724bdb_story.html> Accessed 2020-08-03.

[8] Greninger, A. L., A. Bryan, G. Pepper, et al. 2020. "Performance Characteristics of the Abbott Architect SARS-CoV-2 IgG Assay and Seroprevalence in Boise, Idaho." *Journal of Clinical Microbiology*. Forthcoming.

[9] Public Health Ontario. COVID-19 Seroprevalence in Ontario: March 27, 2020 to June 30, 2020. <https://www.publichealthontario.ca/-/media/documents/ncov/epi/2020/07/covid-19-epi-seroprevalence-in-ontario.pdf?la=en> Accessed 2020-08-08.
